# Supplementary material for: Optical neural engine for solving scientific partial differential equations
Source: Nat Commun. 2025 May 17;16:4603. doi: 10.1038/s41467-025-59847-3 (PMC12085686; doi:10.1038/s41467-025-59847-3)
Supplement: Supplementary file 1 — Supplementary Information [file 41467_2025_59847_MOESM1_ESM.pdf]

Supplementary Information: Optical Neural Engine for Solving Scientific Partial Differential Equations

Yingheng Tang[1*†], Ruiyang Chen[2†], Minhan Lou[2], Jichao Fan[2], Cunxi Yu[3], Andy Nonaka[1], Zhi (Jackie) Yao[1*], Weilu Gao[2*]

[1]Center for Computational Sciences and Engineering, Lawrence Berkeley National Laboratory, Berkeley, CA 94720, USA.
[2]Department of Electrical and Computer Engineering, The University of Utah, Salt Lake City, UT 84112, USA.
[3]Department of Electrical and Computer Engineering, University of Maryland, College Park, MD 20742, USA.

*Corresponding author(s). E-mail(s): ytang4@lbl.gov; jackie_zhiyao@lbl.gov; weilu.gao@utah.edu;
†These authors contribute equally

Supplementary Figures

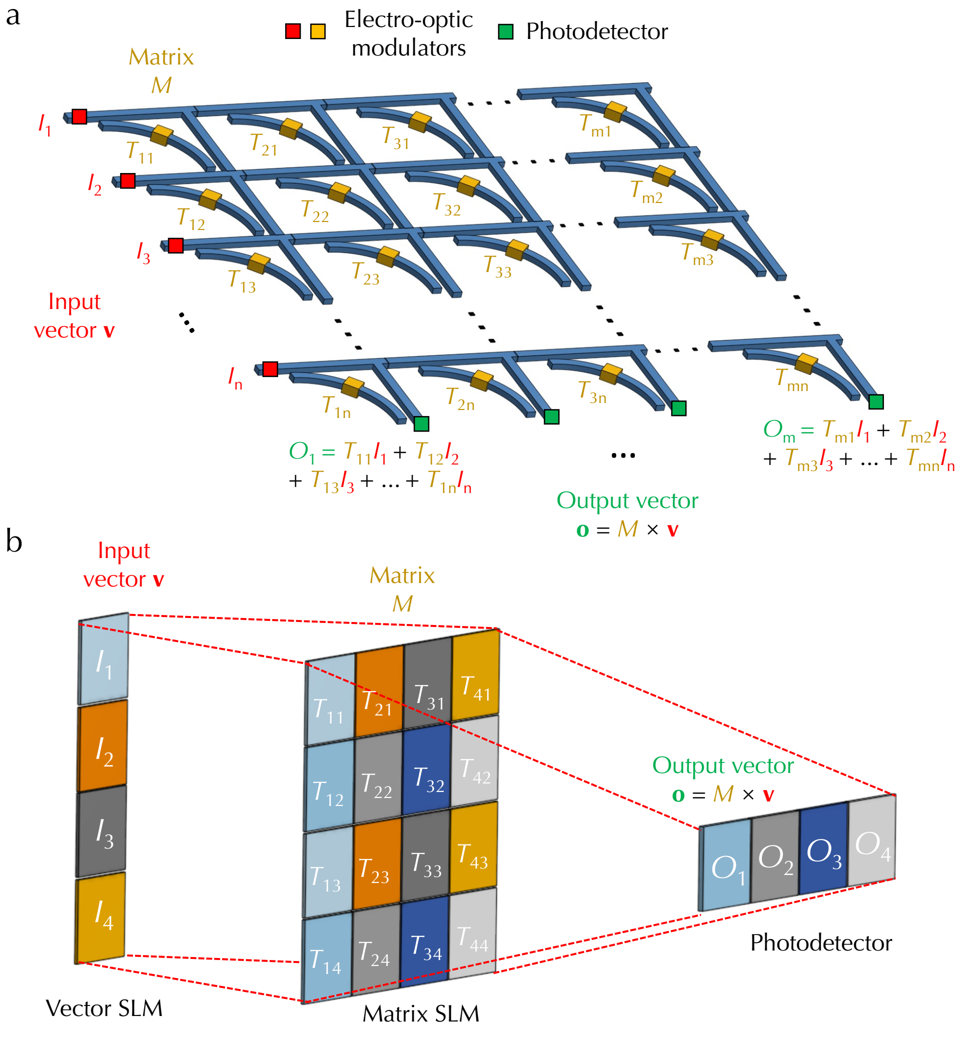

Supplementary Fig. 1 Illustration of optical crossbar structures for performing matrix-vector multiplications in (a) integrated photonic circuits and (b) free space with spatial light modulators (SLMs).

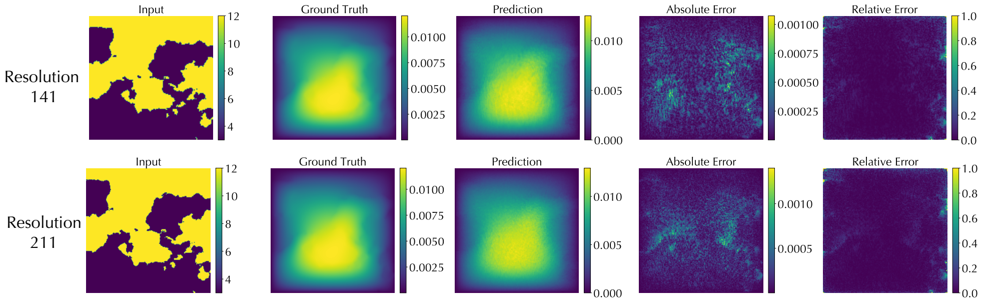

Supplementary Fig. 2 Additional data of solving the Darcy flow equation with the input resolutions of 141 and 211.

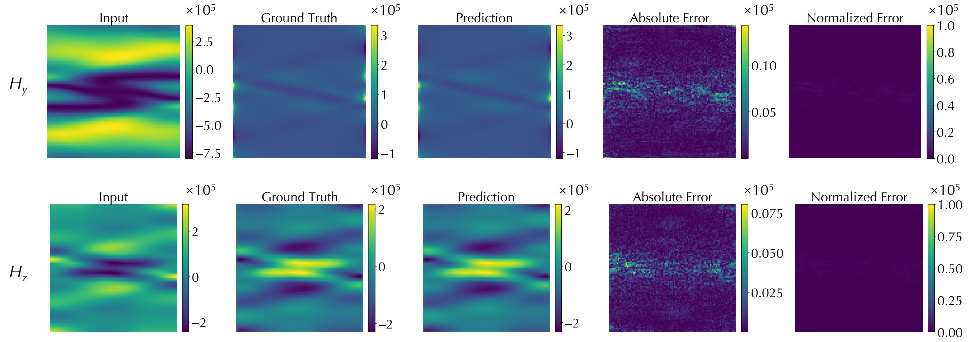

Supplementary Fig. 3 Additional data of solving the demagnetization equation for H_y and H_z components.

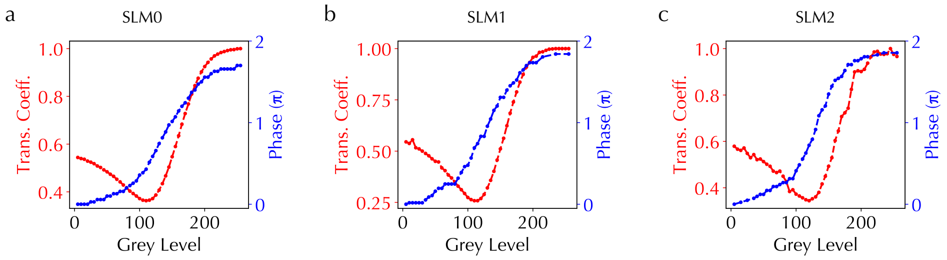

Supplementary Fig. 4 The modulation responses of transmitted electric field amplitude (red lines) and phase (blue lines) in spatial light modulators (SLMs) for (a) encoding input data, (b) first reconfigurable diffractive layer, and (c) second reconfigurable diffractive layer.

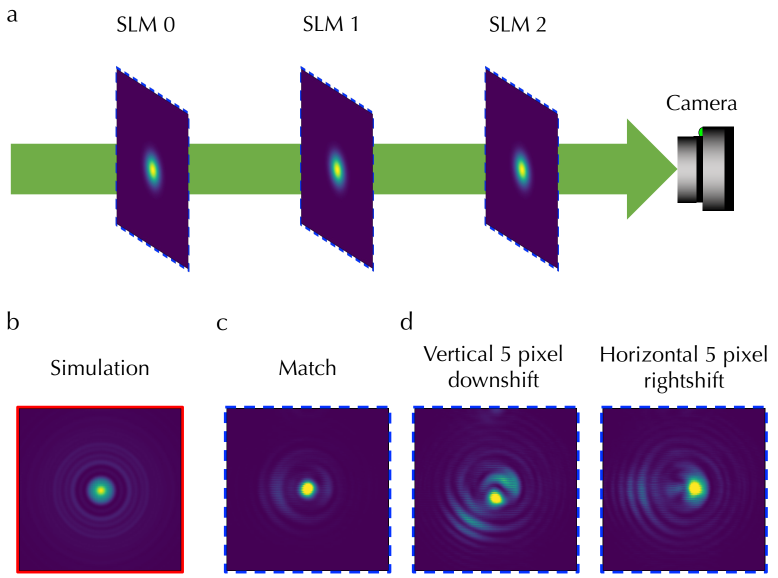

Supplementary Fig. 5 DONN experimental setup alignment. (a) Schematic of loading standard images on input SLM and diffractive spatial light modulators (SLMs). (b) Simulation diffraction pattern, (c) matched experimental diffraction pattern when the optical setup is aligned, and (d) misaligned patterns when load images are shifted horizontally and vertically by five pixels.

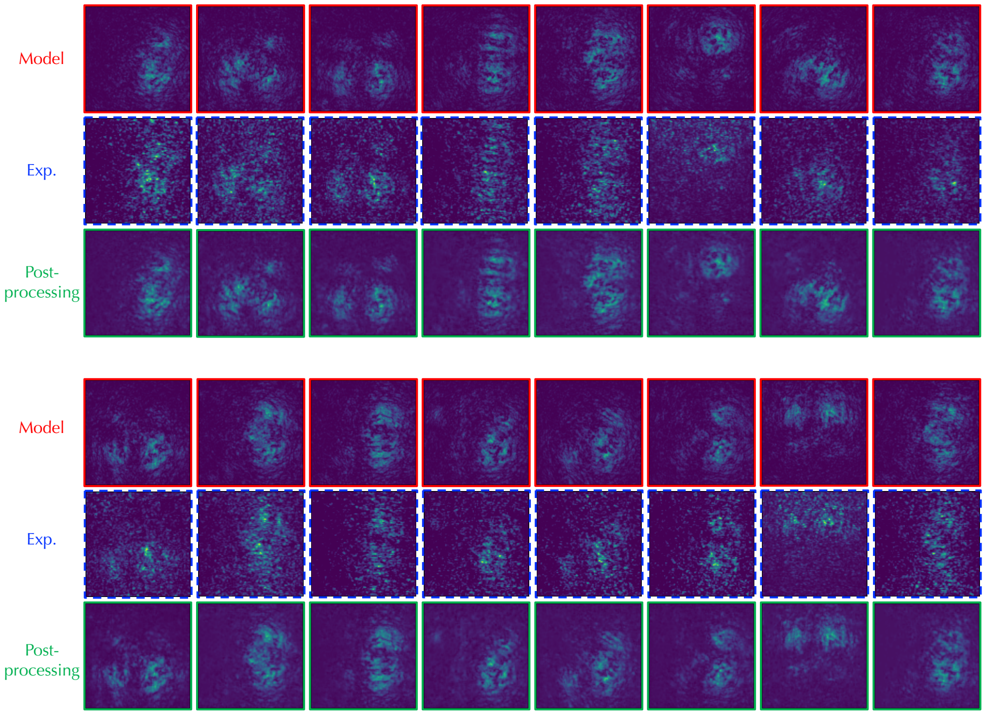

Supplementary Fig. 6 Additional data of experimental measurements for solving Darcy flow equations. Results are obtained from model calculations, experimental measurements (Exp.), and post-processing, respectively.

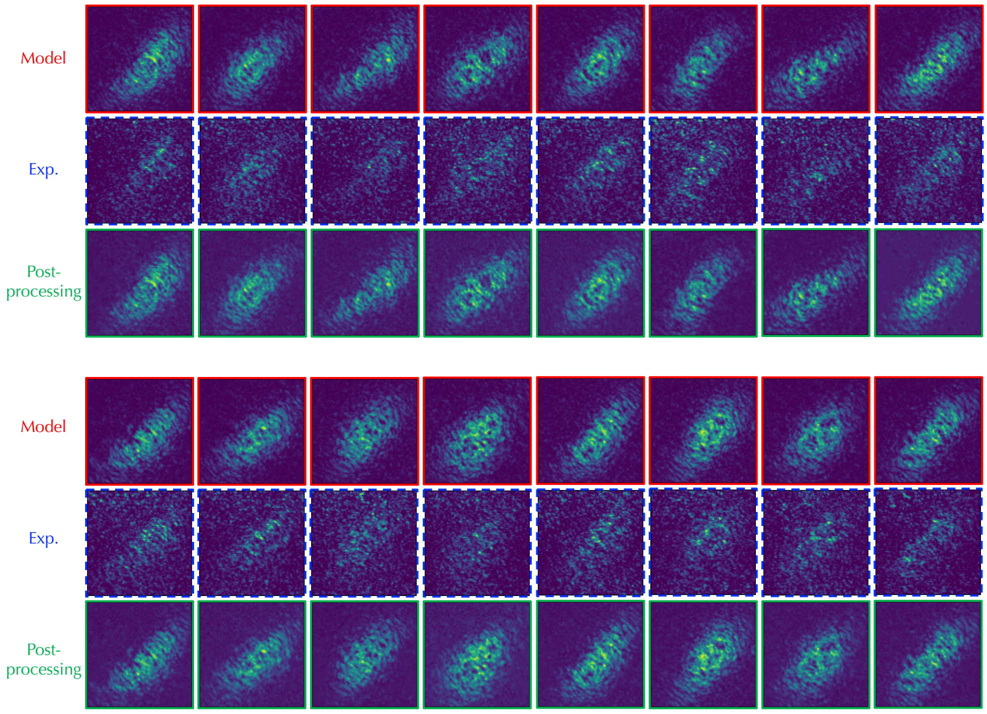

Supplementary Fig. 7 Additional data of experimental measurements for solving Navier-Stokes equations. Results are obtained from model calculations, experimental measurements (Exp.), and post-processing, respectively.

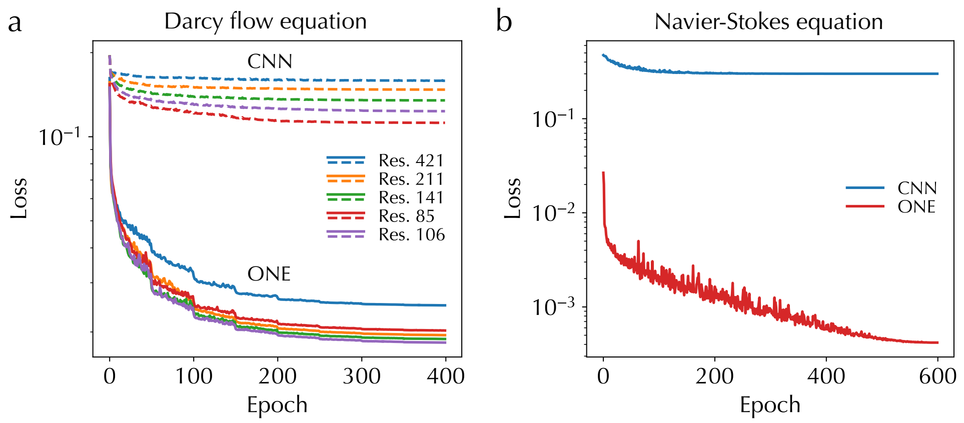

Supplementary Fig. 8 Loss curves for the optical neural engine (ONE) architecture and convolutional neural network (CNN) baseline architecture (a) for solving the Darcy flow equation under 85, 106, 141, 211, and 421 resolutions and (b) for solving the Navier-Stokes equation.

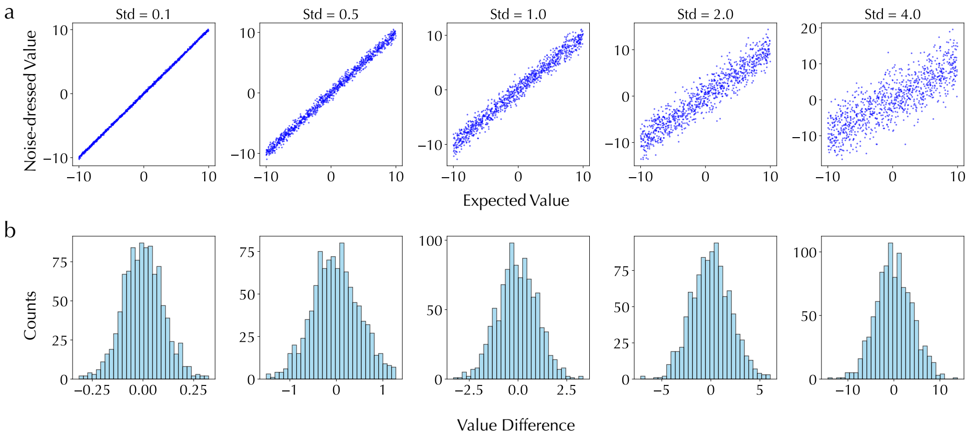

Supplementary Fig. 9 Optical crossbar structures with different standard deviation (Std) values. (a) The noise-dressed value with respect to the expected value and (b) histograms of the difference of these two values under different noise standard deviation levels.

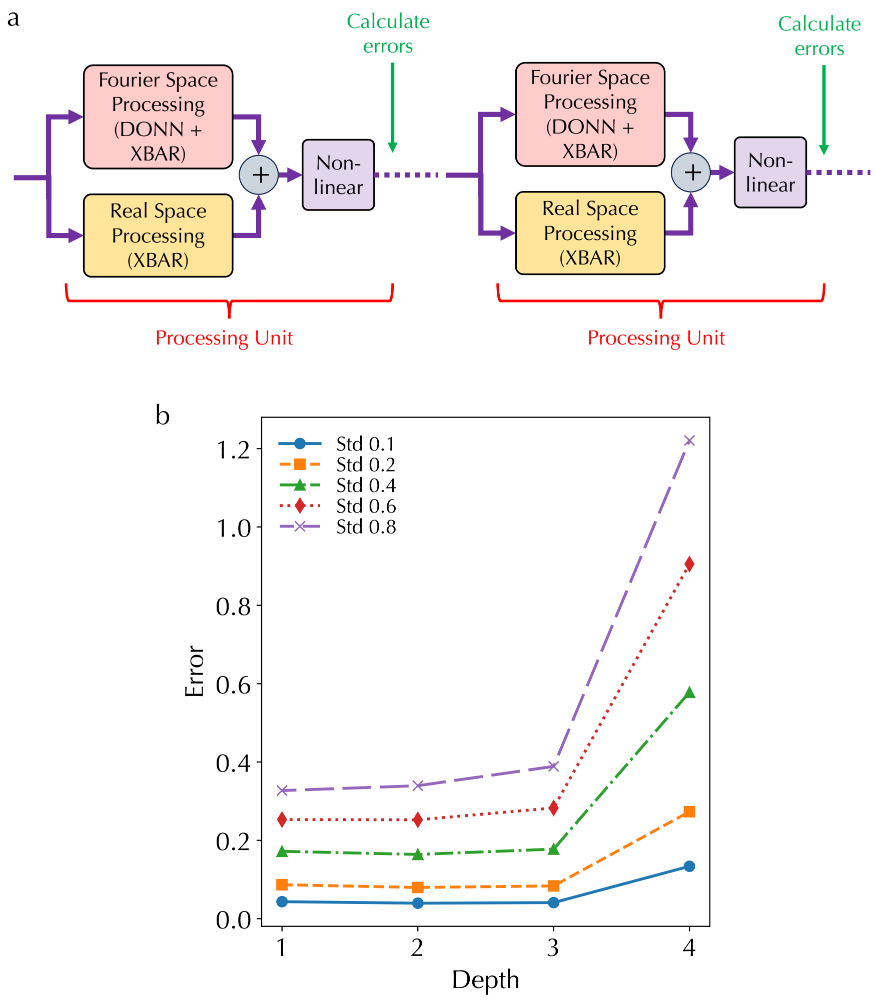

Supplementary Fig. 10 (a) Illustration of calculating intermediate errors in the optical neural engine architecture consisting of diffractive optical neural network (DONN) and optical crossbar (XBAR) structures. (b) Calculation errors as a function of the depth of processing units under various XBAR standard deviation (Std) values.
